# Multi-layered Free-form 3D Cell-printed Tubular Construct with Decellularized Inner and Outer Esophageal Tissue-derived Bioinks

**DOI:** 10.1038/s41598-020-64049-6

**Published:** 2020-04-29

**Authors:** Hyoryung Nam, Hun-Jin Jeong, Yeonggwon Jo, Jae Yeon Lee, Dong-Heon Ha, Ji Hyun Kim, Jae Hee Chung, Young-Sam Cho, Dong-Woo Cho, Seung-Jae Lee, Jinah Jang

**Affiliations:** 1 Department of Creative IT Engineering, Pohang University of Science and Technology, San 31, Pohang, Gyeongbuk Republic of Korea; 2 Department of Mechanical Engineering, Wonkwang University, Iksan-daero, Iksan, Jeollabuk-do Republic of Korea; 3 School of Interdisciplinary Bioscience and Bioengineering, Pohang University of Science and Technology, San 31, Pohang, Gyeongbuk Republic of Korea; 4 Department of Mechanical Engineering, Pohang University of Science and Technology, San 31, Pohang, Gyeongbuk Republic of Korea; 5 Department of Surgery, Collage of Medicine, The Catholic University of Korea, Banpo-daero, Seoul, Republic of Korea; 6 Department of Mechanical and Design Engineering, Wonkwang University, Iksan-daero, Iksan, Jeollabuk-do Republic of Korea

**Keywords:** Biomedical materials, Tissue engineering

## Abstract

The incidences of various esophageal diseases (e.g., congenital esophageal stenosis, tracheoesophageal fistula, esophageal atresia, esophageal cancer) are increasing, but esophageal tissue is difficult to be recovered because of its weak regenerative capability. There are no commercialized off-the-shelf alternatives to current esophageal reconstruction and regeneration methods. Surgeons usually use ectopic conduit tissues including stomach and intestine, presumably inducing donor site morbidity and severe complications. To date, polymer-based esophageal substitutes have been studied as an alternative. However, the fabrication techniques are nearly limited to creating only cylindrical outer shapes with the help of additional apparatus (e.g., mandrels for electrospinning) and are unable to recapitulate multi-layered characteristic or complex-shaped inner architectures. 3D bioprinting is known as a suitable method to fabricate complex free-form tubular structures with desired pore characteristic. In this study, we developed a extrusion-based 3D printing technique to control the size and the shape of the pore in a single extrusion process, so that the fabricated structure has a higher flexibility than that fabricated in the conventional process. Based on this suggested technique, we developed a bioprinted 3D esophageal structure with multi-layered features and converged with biochemical microenvironmental cues of esophageal tissue by using decellularizedbioinks from mucosal and muscular layers of native esophageal tissues. The two types of esophageal tissue derived-decellularized extracellular matrix bioinks can mimic the inherent components and composition of original tissues with layer specificity. This structure can be applied to full-thickness circumferential esophageal defects and esophageal regeneration.

## Introduction

The esophageal tissue refers to the hollow organ between the oropharynx and the stomach, which allows food to pass to the stomach through peristalsis. Congenital or acquired esophageal disorders such as esophageal cancer, malignancy, and esophageal achalasia usually require reconstruction of the defect site after the surgery and stomach, small and large intestine, and skin tissues are used to repair the esophagus tissues^1–3^. Unfortunately, surgical resection and ablation can cause postoperative complications and various surgical morbidities^4–6^. Therefore, a tissue engineering-based approach has been proposed as a promising alternative for reconstruction of circumferential esophageal defects^7–9^.

Human esophageal tissue consists of the mucosa, submucosa, and muscular layers. Therefore, it is necessary to develop esophageal tissueengineering that enables regeneration of esophageal mucosa and muscle layers. Several approaches were considered to fabricate tubular constructs for esophageal tissueengineering. Previous studies have used the porcine bladder-derived dECM sheet^10,11^, collagen sheet^8,12^, and silicone meshes^13,14^. However, these studies focused on fabricating an epithelial layer, and lacked the multi-layered hierarchical structure of the esophagus.

Electrospinning and extrusion-based 3D bioprinting technology have been used to fabricate tubular constructs for esophageal regeneration^14–18^. Electrospinning was considered a promising technology because it can recapitulate microstructures mimicking the environment of the extracellular matrix (ECM) in native tissue. For this reason, electrospinning has been actively applied to esophagus tissue engineering. However, since the use of a rotating mandrel is an essential element for the manufacture of tubular construct, the shape and size of the tubular construct were determined by a rotating mandrel. Thus, technical limitations exist that preclude composition of free-form constructs such as the esophageal mucosa and muscle layers.

Extrusion-based 3D bioprinting has been extensively studied to enable the production of the tubular free-form construct in tissue engineering^19,20^. This technology can produce free-form structures, unlike electro-spinning technology. We proposed and fabricated a unique 3D printing method called by the dragging technique. The dragging technique uses the stretching phenomenon when viscoelastic material is dispensed throughout the printing nozzle. It is unique easy-to-control method of bioprinting to create more flexible and complex 3D construct by changing the line width during one printing path. The main advantage of this printing technique is that it not only enables to produce of free-form multi-layered construct, but also can manufacture an adjustable porous structure. In this study, we applied this technique to develop the Multi-layered Free-form porous Tubular (MFT) construct for esophageal tissue engineering to demonstrate structural fidelity as well as flexibility.

Suitable bioinks for fabricating cell-laden constructs have been developed to provide proper conditions to synthesize ECM properly for tissue development and maturation. The cells in the 3D construct can be affected by the combination of biophysical or biochemical signals exerted by cell–cell interactions^21,22^, growth factor^23,24^ and ECM (e.g., collagen, gelatin, fibrin)^25–27^. Collagen is used as a general source as a printable material because of its high biocompatibility and functionality. Originally, ECM in the body has a very complex network, in which various proteins such as collagen, elastin, and GAGs are composed, and their physical and chemical compositions differ between tissues^28^. For this reason, decellularization materials with the greatest similarity to actual tissues began to be developed^29^. Researchers developed a technique for successful decellularization of various tissues (e.g., heart, cartilage, adipose, pancreas, blood vessel, esophageal tissues) and 3D bioprintingwith them through previous studies^30–34^.

In this study, we developed a extrusion-based 3D printing technique to control the size and the shape of the pore in a single extrusion process, so that the fabricated structure has a higher flexibility than the conventional process. Based on the suggested technique, we developed a bioprinted 3D esophageal structure with multi-layered features and converged with biochemical microenvironmental cues of an esophageal tissue by using decellularizedbioinks derived from mucosal and muscular layers of native esophageal tissues. The dECMs are printed into the MFT construct, and we thereby obtainedpore-controllable multilayered free-form tubular constructs with bioink that mimic each esophageal layer’s microenvironment. The manufactured structures were confirmed as suitable for esophageal regeneration constructs through *in vitro* experiments.

## Results

### Development of a 3D printing method

We developed a 3D printing method (called dragging technique) for fabricating multi-layered free-form tubular constructs by using the stretching phenomenon of viscoelastic material. This technique utilizes the same structural and technical characteristics to the widely applied extrusion-based 3D printing, but it can allow a printed structure to obtain altered line width and pore size in one extrusion step. For example, when printing a porous layer thinner than nozzle diameter, it is impossible to produce pores by the conventional method,while the dragging technique can be used to selectively create adjustable pores on the thin layer surface (Supplementary Fig. 1a). Consequently, the dragging technique can fabricate a thin porous polymeric layer and as well as manufacturing any free-form shapes. The mechanism of the dragging technique controls the stretching of extruding viscoelastic at the exit of the printing nozzle to build a 3D structure. First of all, the dragging design process has to essential performed for using dragging technique printing, after a design of the 3D shape to be built. The key point of the dragging design process is the column design for stretching printing material and regulating the distance between columns, which allows controlling the size of the pores (Supplementary Fig. 1b).

**Figure 1:**
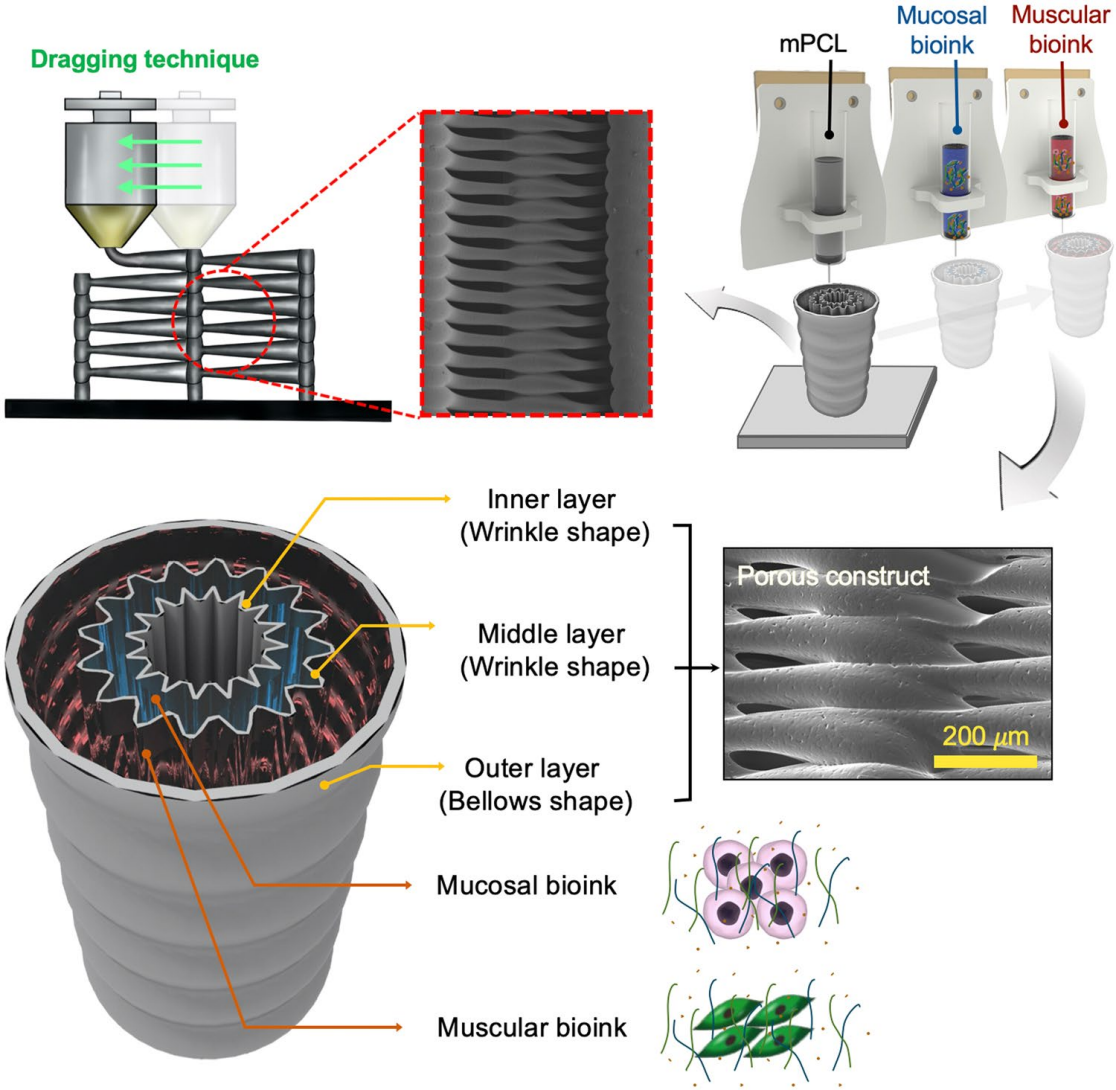
Schematic illustration of the biomimetic esophagus scaffold. Multilayered free-form 3D cell-printed tubular construct using dragging technique with decellularized inner and outer esophageal tissue-derived bioinks (mucosal bioink:emuc-dECM with hEECs, muscular bioink: ems-dECM mixed with hESMCs).

We designed two representative shapes to mimic the multi-layered architectures of native esophagus tissue. The outer layer of the MFT construct was designed in a bellows shape to endure external stresses applied under peristaltic movement when eating food and the surgical operation (Fig. 1). The inner and middle layers of the MFT construct were designed in a wrinkled shape to mimic the esophageal mucosa layer. In addition, we fabricated 3Dprinted MFT constructs with similar structural morphology to the native esophagus (Supplementary Fig. 2).

### Fabrication and analysis of MFT constructs

**Figure 2:**
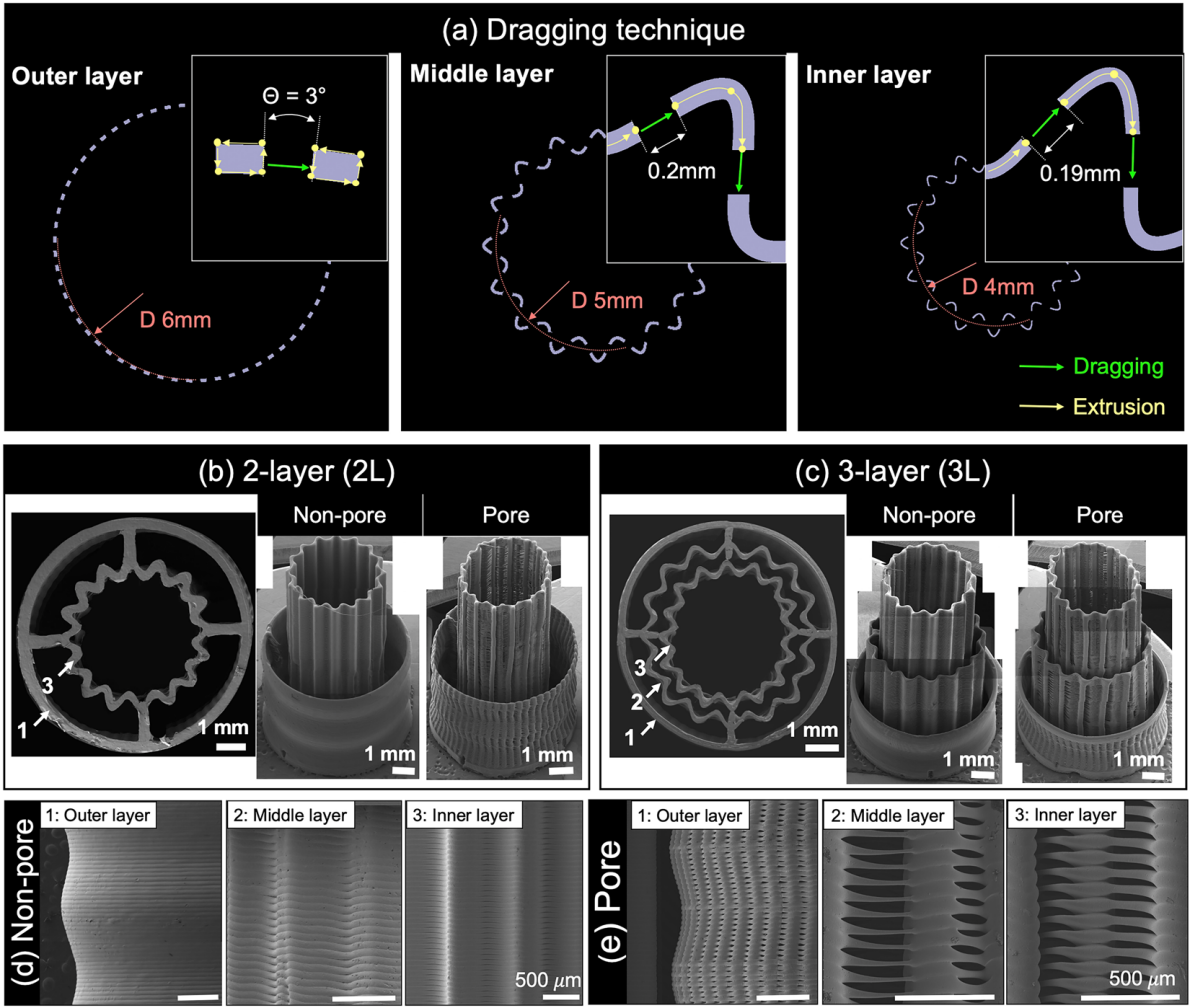
Schematic illustration of the dragging technique and morphological analysis of the 3D printed MTF construct. (**a**) The parameters of the dragging technique. FE-SEM images of (**b**) 2-layer pore/non-pore top view (left) and isometric view (right) and (**c**) 3-layer pore/non-pore top view (left) and isometric view (right). Morphological analysis of FE-SEM image of (**d**) 3-layer non-pore, and (**e**) 3-layer pore.

To fabricate MFT constructs, all layers were designed according to the native tissue architectures, and we generated specific g-code using an in-house build code generator for the dragging technique. The angle and distance of dragging regions, the feed rate, and air pressure are important parameters for applying the dragging technique. By changing the angle and distance parameter of the dragging region, the size of pore on the tubular constructs can be controlled. For the sequential pore size printing, the outer layer of the dragging region was designed with 3-degree gaps, and the middle and inner layer were designed with 0.2 mm and 0.19 mm gaps, respectively (Fig. 2a).

The morphological characteristics were examined by FE-SEM images. We clearly observed the presence of precise wrinkles in the vertical-direction at the middle and inner layers (2 and 3, respectively). The radii of the center circle at wrinkles of inner and middle layers were fabricated by 4 mm and 5 mm, respectively (Fig. 2b,c). In the isometric-view images of the 2-layered (2 L) construct, it was confirm that the existence of pores was clearly different depending on use of the dragging technique (Fig. 2b). Similarly, the 3-layered (3 L) construct showed clear existence of a wrinkle shape and pores at the surfaces of inner and middle layers (Fig. 2c). The side-view of FE-SEM images showed the precise bellows shape of 3-layered construct (Fig. 2d,e). The non-pore (NP) groups of the MFT construct showed all layers stacked with medical grade PCL (mPCL) strands without pores (Fig. 2d). On the contrary, the Pore(P) group of the MFT construct that used a dragging technique showed clear existence of periodic pores at each layer (Fig. 2e). The size of mPCL strands at the inner and middle layers and the outer layer were around 100 μm and 170 μm, respectively. Average pore sizes of each layer are given in Table 1.

**Table 1:** Measured pore sizes of the each layer.

(*μ*m)	Outer layer	Middle layer	Inner layer
Width	89.33 ± 11.07	341 ± 18.38	252.5 ± 0.78
Height	27.77 ± 6.68	49.33 ± 11.18	38.15 ± 1.22

### Simulation for validating mechanical flexibility of the MFT construct

**Figure 3:**
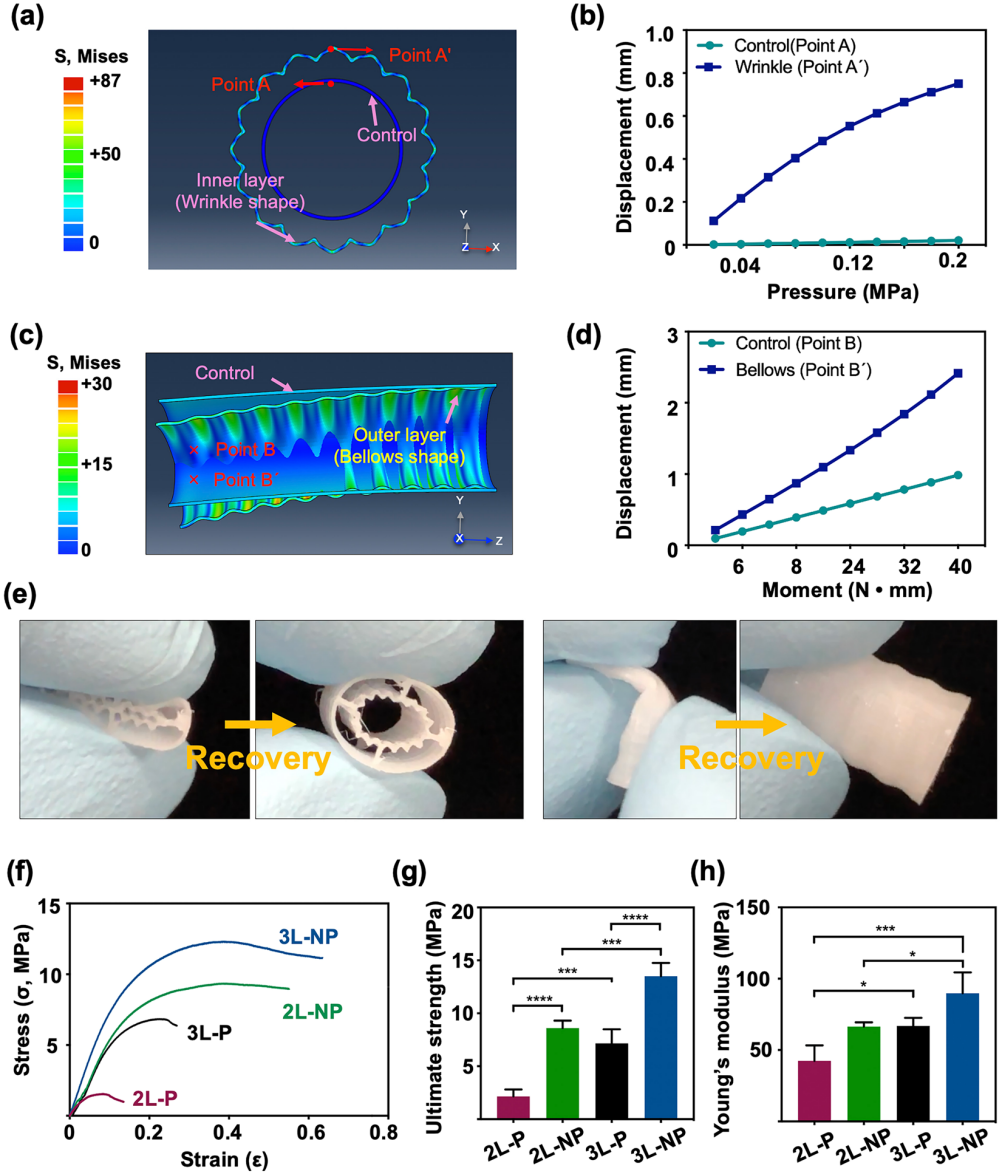
Computational and experimental analysis of the mechanical characteristics of the winkle and the bellows shapes. (**a**) Deformed configuration and radial-directional pressure (0.2 MPa) contour plot, (**b**) Comparison of pressure result at each point A, A′, (**c**) Deformed configuration and uniform bending moment (40 N•mm) contour plot, (**d**) Comparison of bending moment result at each point B, B′, (**e**) Photographs of recovering test at 3D printed of 2-layer pore construct, (**f**) Stress-strain responses of the scaffolds (2-, 3-layer pore/non-pore), (**g**) Comparison of ultimate strength, (**h**) Comparison of Young’s modulus (*p < 0.05, ***p < 0.0005, ****p < 0.0001).

Finite element analysis (FEM) was performed to investigate the effect of wrinkles and bellows shape to determine the mechanical flexibility. The models of hollow cylindrical shape with wrinkles and bellows shape were prepared for simulation (Fig. 3a,c). For comparison to the MFT construct, the plane hollow cylindrical model was prepared as a control group. The relevant data of boundary conditions are described in Supplementary Fig. 3.

For the simulation of radial-direction flexibility of inner and middle layers with wrinkles, uniform pressure (0.2 MPa) was applied at the inner surface of the wrinkle model and the control model (Fig. 3a). The radial-direction flexibility at the same pressure was significantly more deformed with the wrinkle model than with the control model. Quantitative y-directional displacement at the same node point(A and A´) in each model was predicted as 0.02 mm and 0.75 mm, respectively (Fig. 3b).

To simulate the bending flexibility of outer layer with bellows shape, uniform bending moment (40 N·mm) was applied at the center point of the bellows model and the control model (Fig. 3c). Under the bending moment, the predicted deformation showed more flexible movement in the bellows model. Quantitative displacement at center circle node point in each model (B and B′) was predicted as 0.98 mm and 2.41 mm, respectively (Fig. 3d).

In terms of the simulation results, the proposed method can be expected to have a flexible physical property with respect to the external stress by the specific characteristic such as wrinkle and bellows shapes. If these flexibility features are applied to esophageal tissue engineering constructs, they can be expected to serve effectiveroles in peristalsis and physiological stress in esophagus tissue.

### Mechanical property analysis of 3D printed tubular constructs

Several mechanical tests were performed for comparison with respect to the pore’s present and the number of layers in the 3Dprinted MFT structure.

First of all, simple radial-direction compression and bending with bare hand was performed. For the 3Dprinted 2L-P MFT construct, the shape was recovered to original state without break down after applying compression and bending force (Fig. 3e).

Based on the stress–strain curves, the ultimate strength of each case of the MFT construct (2L-P, 2L-NP, 3L-P, and 3L-NP) was calculated as 2.16 ± 0.6 MPa, 8.60 ± 0.7 MPa, 7.15 ± 1.3 MPa, and 13.50 ± 1.3 MPa, respectively (Fig. 3f,g). The Young’s modulus of each case (2L-P, 2L-NP, 3L-P, and 3L-NP) was calculated as 25.32 ± 10.02 MPa, 66.43 ± 2.97 MPa, 66.94 ± 5.6 MPa, and 86.86 ± 14.63 MPa, respectively (Fig. 3h). In addition, ultimate strengths of the MFT constructs with or without dECM gel (2L-P, 2L-P with dECM gel, 3L-P, 3L-P with dECM gel) were calculated as 2.43 ± 0.45 MPa, 3.76 ± 0.07 MPa, 6.92 ± 1.34 MPa, 7.20 ± 0.1 MPa, respectively (Supplementary Fig. 4).

### Decellularization of the esophageal tissues and their biochemical analysis

**Figure 4:**
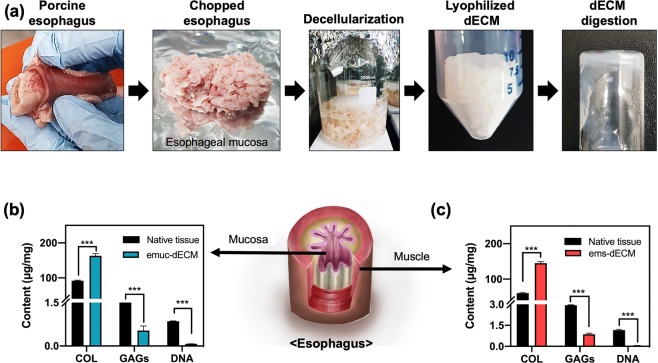
Preparation and characterization of the esophageal bioinks. (**a**) Decellularization process of the mucosal and muscular esophagus, (**b**) Component of the esophageal mucosal dECM, (**c**) Component of the esophageal muscular dECM (***p < 0.0005).

To mimic the components and compositions of each esophageal layer, we divided the esophagus into two parts: the mucosa and the muscular parts. Each part was decellularized by a physical and chemical process (Fig. 4a). After this process, obtained the porcine esophageal mucosa-derived decellularized extracellular matrix (emuc-dECM) and porcine esophageal muscular-derived decellularized extracellular matrix (ems-dECM).

The efficiencies of these developed decellularization methods were evaluated by DNA quantitative analysis. After the decellularization, most of DNA was removed, with only 0.073 ± 0.004 μg/mg remaining emuc-dECM and 0.042 ± 0.016 μg/mg ems-dECM DNA contents (Fig. 4b,c). The emuc-dECM residual DNA value is between those of the two commercially products Alloderm^TM^ (0.95 μg/mg) and Permacol^TM^ (0.063 μg/mg)^35^. The ems-dECM residual DNA value is under 0.05 μg/mg. It could be said that there will be no immune rejection if the residual DNA is less than 0.05 μg/mg^34^. Thus, we could successfully decellularize mucosal and muscular parts of the porcine esophageal tissue.

Representative ECMs including collagen and glycosaminoglycans (GAGs) were also quantified by using hydroxyproline assay and dimethylmethylene blue assay, respectively. Results showed that, after decellularization, the remaining collagen steeply increased and the remaining GAGs reduced moderately (Fig. 4b,c). In general, the concentration of collagen per unit volume seems to increase proportionately because the decellularizationprocess removes cellular components from the esophagus tissue parts. On the other hand, because of chemicals such as detergents used in decellularization, some glycoproteins and GAGs are inevitably attacked. Thus, remaining GAGs reduced moderately.

### Rheological and biological analysis of emuc- and ems-dECMbioinks

**Figure 5:**
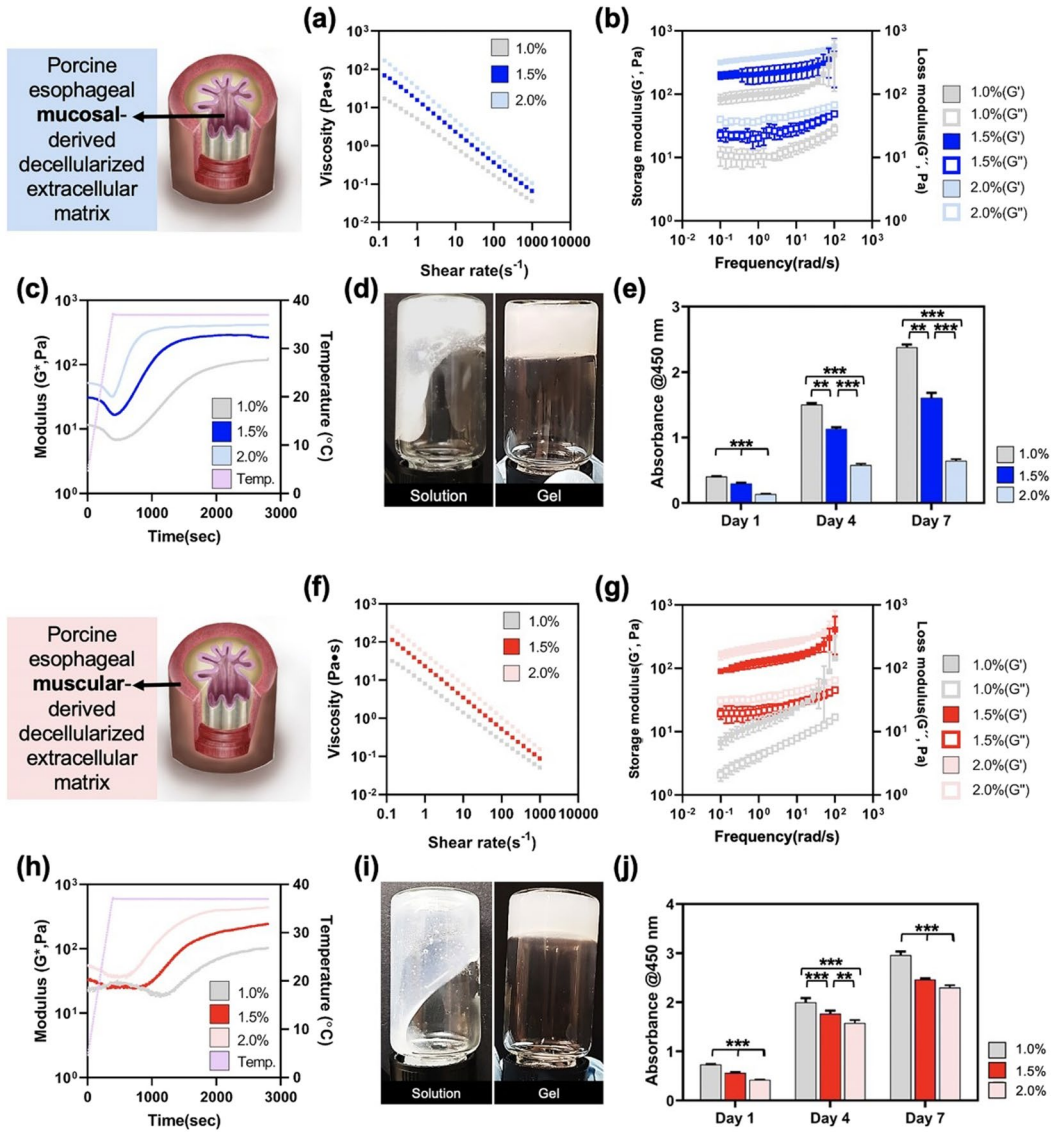
Rheological behavior for emuc- and ems-dECM bioinks of various concentrations and their effects on cell proliferation. Viscosity of emuc- (**a**) and ems-dECM bioinks (**f**) measured at 4 °C. Dynamic modulus of emuc- (**b**) and ems-dECM bioinks (**g**) at various frequencies at 37 °C. Gelation kinetics of emuc- (**c**) and ems-dECM bioinks (**h**) at temperatures varying from 4 to 37 °C, and images of the sol-gel transition (**d**,**i**). Cell proliferation analysis for (**e**) emus-dECM encapsulated with human esophageal epithelial cells and (**j**) ems-dECM encapsulated with human esophageal smooth muscle cells. (**p < 0.005, ***p < 0.0005).

Rheological and biological analysis was conducted to investigate the characteristics of emuc- and ems-dECM bioinks and to select the optimal bioink concentration for further study. We measured the viscosity of dECM pre-gel solutions at 4 °C (Fig. 5a,f) to simulate 3D bioprinting conditions. Generally, hydrogels with a high viscosity enhance printability, and printable biomaterials exhibit shear-thinning behaviorfor all shear rate range. The results demonstrated that the emuc-dECM and ems-dECM pre-gel solutions had higher viscosity at higher concentrations. The storage and loss moduli of the dECM gels were also measured at 37 °C (Fig. 5b,g). Most of the esophageal dECM pre-gel solutions were crosslinked at the initial time point, and dECM exhibited a higher storage modulus than loss modulus in the 1.5 and 2.0% bioinks. This result indicates that the both 1.5 and 2.0% dECM bioinks can provide a stable and reinforced substrate under dynamic conditions. In contrast, the storage modulus of the 1.0%emuc-dECM and ems-dECM bioinks was unstable at high frequencies. Gelation kinetics in the 1.5 and 2.0% emuc- and ems-dECM pre-gel solutions were clearly observed at a physiologically relevant temperature (37 °C; Fig. 5c,h). However, gelation within the 1.0% solution took longer compared to those at higher concentrations (Fig. 5d,i).

The optimal concentration of each dECM bioink was investigated by culturing esophageal epithelial cells and smooth muscle cells and observing their proliferation rate over 7 days (Fig. 5e,j). The cell proliferation rate was higher at a 1.0% concentration than at 1.5 and 2.0 % for both the emus-dECM and ems-dECM bioinks. However, we selected the 1.5 % concentration because of its structural stability under dynamic conditions.

### Study of cell behavior in the 3D cell-printed tubular constructs

**Figure 6:**
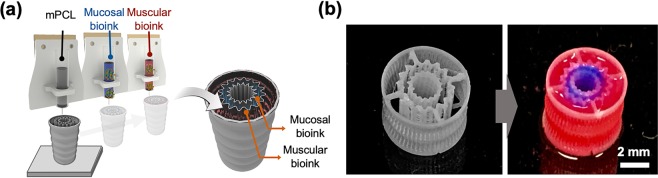
Fabrication of the multilayered free-form tubular (MFT) construct with the inner and outer bioinks. (**a**) Fabrication process by 3D bioprinting. (**b**) The picture of the MFT construct only and the bioink injected MFT construct. Mucosal bioink stained by blue ink and muscular bioink stained by red dyes for the distinction.

We fabricated a 3D cell-printed tubular construct using cell-laden emuc- and ems-dECM bioinks (Fig. 6a**;** Supplementary Video 1). The figure shows the original multilayered free-form tubular (MFT) structure constructed using mPCL both immediately after fabrication and following the sequential printing of the emuc-dECM and ems-dECM bioinks between the layered components of the MFT structure (Fig. 6b). To visualize the bioinks clearly, blue and red-colored food dyes were added to the emuc- and ems-dECM bioinks, respectively. Although the inside of the MFT structure was porous, the blue and red colors did not mix.

**Figure 7:**
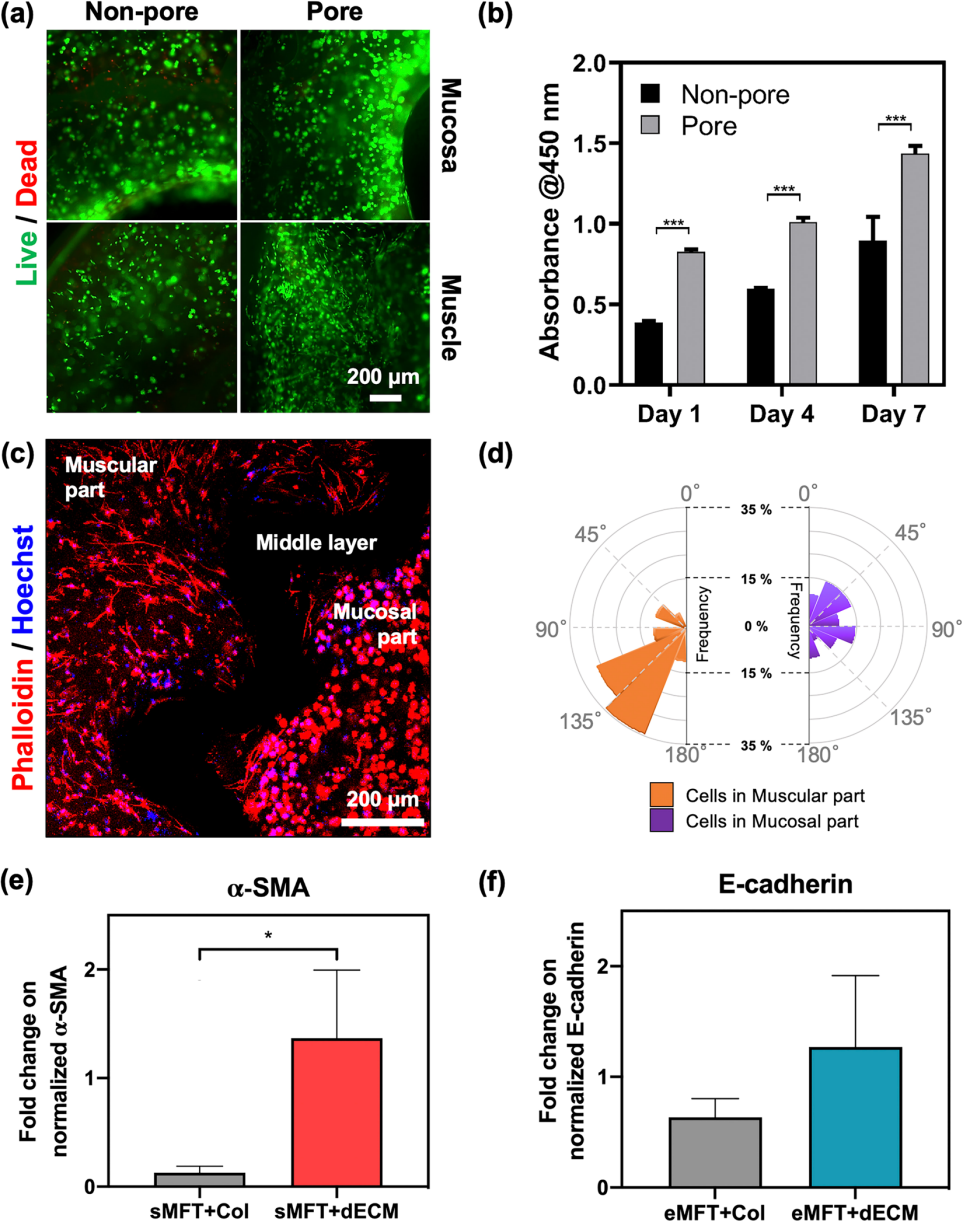
*In vitro* studies with multilayered free-form tubular (MFT) construct with the inner and outer bioinks. (**a**) Live/dead assay of the MFT construct with non-pore and pore at day 7. (**b**) CCK-8 analysis of the non-pore and pore construct. (**c**) Fluorescence staining of the MFT construct. (**d**) The analysis graph of the cell morphology in (**c**). Real-time PCR analysis results of the (**e**) alpha-smooth muscle actin (α-SMA) and (**f**) E-cadherin. eMFT+dECM is hEECs-laden emuc-dECM bioink, eMFT+col is hEEC-laden collagen bioink, sMFT+dECM is hESMCs-laden ems-dECM bioink and sMFT+col is hESMCs-laden collagen bioink (*p < 0.05, ***p < 0.0005).

To investigate the effect of porosity on cell survival, we fabricated 3D cell-printed tubular constructs with and without pores. Cells in the printed constructs were cultured for 7 days and their viability and growth rate assessed using live/dead staining and CCK-8 assays, respectively. Most of the cells in in both the mucosa and muscle sections of the porous construct were alive (Fig. 7a). In contrast, the number of dead cells between the middle and outer layers of the non-porous construct was higher, which was in accordance with the cell proliferation results, while the number of green cells was much higher in the porous than in the non-porous construct. The growth rate also differed significantly between the porous and non-porous constructs over the 7-day culturing period (Fig. 7b). The proliferation of both cell types in the porous construct was more than 1.7-fold higher than that in the non-porous construct, illustrating that the porous construct improves both cell proliferation and viability after the printing process.

The morphology of the cells in the mucosal and muscular sections clearly differed (Fig. 7c), which indicates that the two cell types remained separate and interacted with the extracellular matrix bioink to bind to specific receptors and remodel their environment. The cells in the mucosal section were rounded and those in the muscular section were elongated, thus reflecting the standard morphology of each cell type. To quantify the cellular morphology, the orientation of the cells was analyzed using image processing (Fig. 7d). It was found that the esophageal mucosal epithelial cells had a cobblestone (epithelium-like) morphology, while the muscular cells exhibited a classical mesenchymal morphology. The two cell types also differed in terms of their distribution in the 3D printed construct, with the cells in the 3D emuc-dECM bioink exhibiting a homogenous distribution with an epithelium-like morphology and those in the 3D ems-dECM bioink showing a unidirectional alignment with a spindle-like shape.

We examined the gene expressions of alpha-smooth muscle actin (α-SMA) as hSMCs-specific marker and E-cadherin as hEECs-specific marker to analyze the functions of esophageal dECM and collagen in the MFT construct. α-SMA was highly expressed in esophageal dECM comparison with collagen in the inner layer (Fig. 7e). Also, E-cadherin was expressed in esophageal dECM better than collagen in the outer layer (Fig. 7f).

## Discussion

In the present study, we proposed a 3D bioprinted MFT construct that has a similar external and internal morphology to native esophagus tissue. The dragging technique and tissue-specific bioinks are the key to successfully developing a strategy for esophageal tissue reconstruction.

Several biofabrication approaches, including 4-axial printing and electro-spinning with a rotating rodand transplantation of the heterogeneous decellularization organs have been used to fabricate multi-layered tubular constructs^14–18,20,36,37^. However, these technologies require the use of a rotating metal rod of the same size as the diameter of the structure, meaning that the production of constructs with various dimensions is difficult. In addition, freedom in shaping the tubular structure in the vertical direction is limited. On the other hand, the dragging technique enables the manufacture of a hollow multi-layered structure with a free-form shape; it particular, it allows the production of tubular structures with an adjustable line width and pore size. However, dragging technique has the disadvantage that its building material must have the self-supporting property. In addition, because the stretching properties vary depending on the viscosity of the material, there are differences and limitations in pore size control for each self-supporting material.

Pores in scaffolds employed in tissue engineering are essentialfor effective cell maturation because they facilitate the supply of nutrients and promote intercellular interactions. A porous MFT with inner, middle, and outer layers that can mimic the wrinkled morphology of the mucosa layer and a bellows shape that improves flexibility can be fabricated using the dragging technique. Also, morphological analysis revealed that each layer of the MTF construct was porous. To effectively supply nutrients and maximize the interaction between hEECs and hESMCs, the pores of the inner and the middle layers were designed to be larger than those of the outer layer, with the pores in the outer layer designed to supply a steady rate of nutrients and to minimize the growth of other soft tissue on the outside. When cell proliferation was analyzed, the MFT structure with pores exhibited a higher cell proliferation rate in all sections than the non-porous MFT structure. These results thus demonstrate that pores provide an environment that is more conducive for cell proliferation.

The mechanical flexibility of the MFT structure depending on its structural features was quantitatively analyzed using simulations. When the *y*-direction deformation of points A and A’ was compared, the structure with wrinkles in the vertical direction allowed 35-times greater displacement. When pressure what applied to the inner surface of the hollow cylindrical model in the radial direction, the structure withwrinkles allowed more flexible movement in response to external stimuli.When a uniform bending moment was applied to the center point, the hollow cylindrical model with a bellows shape exhibited 2.5-times greater displacement. Consequentially, we quantitatively predicted that the wrinkles and bellows shape, which mimic the morphology of the esophagus, of an esophageal tissue-engineered substrate with peristaltic properties would improve flexibility, provide structural safety, and be more suitable for the physiological environment.

When compression in the radial direction and a bending force were applied by hand to the fabricated 2L-P construct, the shape recovered. For the 3L-P construct, the shape recovered after external stress without collapsing or breaking (Supplementary Video 2). These results indicate that the physiological stress that the esophagus is subject to falls within the elastic region of the mPCL. Therefore, the fabricated MFT structure is expected to be able to effectively adapt to esophageal movements such as peristalsis in animal experiments.

Ideally, a tissue-engineered scaffold should have mechanical properties similar to the original tissue or organ. As such, when faced with repetitive movements such as peristalsis, the mechanical properties of esophageal tissue-engineering substitutes should be similar to the original esophageal tissue otherwise the substitute may leak or rupture after implantation. The maximum tensile stress of the human esophagus is 1.2 MPa^38^. In the present study, the ultimate strength of the 2L-P was found to be 2.16 ± 0.64 MPa, which is similar to human esophagus tissue. The ultimate strength of 3L-P was 7.15 ± 1.3 MPa, which is sufficient to withstand physiological stresses after transplantation. As a result, the MFT structure fabricated in this study can be designed to display the optimal mechanical properties by varying the number of layers and the size and number of pores.

The development of dECM for specific types of esophageal tissue (i.e, emuc-dECM and emc-dECM) enables the multilayered esophagus to be mimicked. A well-known property of dECM is that it effectively mimics the micro-environment around a cell in specific tissue^21,29^. The ECM microenvironment plays an important role in directing and mediating a variety of cell behaviors, including adhesion, proliferation, growth, and differentiation^22–24^. By providing each cell with a suitable dECM layer, it is possible to obtain an optimal environment to direct the cells’ behavior^39–42^.

By analyzing the rheological properties of the dECMs and applying these to the cells, we were able to determine the optimal bioink concentration for the proposed scaffold. It was found that a concentration of 1.5% was most effective in promoting cell survival and growth based on the relationship between the emuc-dECM and hEECs and between the emc-dECM and hESMCs. We injected the bioinks at a concentration of 1.5% into each layer using 3D printing. This allowed us to obtain a multilayered scaffold with an inner layer consisting of hEECs with a suitable emuc-dECM and an outer layer consisting of hSMCs with a suitable emc-dECM, as in a real esophagus. In addition, the injected cells were uniformly distributed within the construct, which is a major advantage of 3D bioprinting^43^.

An important consideration for the proposed scaffold was whether it was possible for important media and oxygen to be transported into the multilayers. To confirm this, porous and non-porous MFT structures were produced using the dragging technique and their performance compared with *in vitro* experiments. Using CCK-8 assays, it was clear that the porous scaffold was more effective than the non-porous scaffold in terms of cell proliferation. Similarly, in the live/dead assays, we observed a higher number of dead cells and fewer live cells in the non-porous scaffold, with a number of dead cells found at the interface between the inner and outer layers. Also, through the analysis of the gene expression, we confirm the MFT construct+esophageal bioinks improved the function of hEECs and hSMCs.

The advantages of MFT construct with bioink were clearly confirmed in the *in vivo* maturation experiments. We confirmed a better relationship between the *in vivo* cells and the bioink in the MFT construct with bioink (Supplementary Fig. 5). Based on this approach, the MFT construct with bioink appears to be a promising alternative strategy for the reconstruction of circumferential esophageal defects.

## Conclusion

In this paper, we have designed and fabricated an innovative artificial esophageal construct that mimics the native esophagus using 3D bioprinting. We fabricated an advanced multilayered construct that is morphologically similar to the original esophagus using the dragging 3D printing technique. Decellularizedbioinks from mucosal and muscular layers of the esophagus were then developed to produce a structure that demonstrated the required cell specificity to accurately recreate the esophagus. Using *in vitro* experiments, it was confirmed that the fabricated construct had a porous multilayered structure that promoted cell proliferation, verifying its suitability as an esophageal construct.

The MFT construct developed in this study is a very promising approach that can be applied to the treatment of full-thickness circumferential esophageal defects and expected to be suitable for esophageal regeneration. In the future, we plan to apply the MFT construct to confirm its suitability as a clinical esophageal construct through an animal test.

## Methods

### Structure characterization

A multilayered free-form tubular (MFT) construct was designed using a commercial Computer Aided Design (CAD) program (CATIA R13 V5, DassaultSystèmes®, France). To mimic the mucosal shape of the esophagus, the inner and middle layers of the MFT structure were designed to have wrinkles, while the outer layer was designed to have a bellows shape in order to effectively withstand the physical stresses associated with the surgical and physiological environments. The inner and middle layers had center circle diameters of 4 mm and 5 mm, respectively, while the diameter of the outer layer was 6 mm. The geometrical features of the MFT structure are illustrated in Supplementary Fig. 6.

FE-SEM (S-4800, Hitachi, Japan) was used to observe the morphology and porous structure of the MFT construct produced by mPCL without bioinks. The mPCLMFT structure was coated with platinum for 60 seconds before FE-SEM analysis. SEM images were captured using a beam intensity of 5.0 kV.

### Fabricated of the multilayered esophageal tubular structure using 3D bioprinting

The MFT construct was fabricated using medical-grade PCL (mPCL, Resomer C209, Evonik, Germany). Molecular weight of mPCL was 73,000 and inherent viscosity is 0.8–1.0 dl/g (0.1% in chloroform, 25 °C). A lab-made precision multi-head pneumatic printing system was used to carry out the 3D printing. To print the MFT construct, mPCL pellets were melted in a stainless steel print head (SS10, U-Jin Tech., Korea) at 85 °C and keep the print bed at 35 °C while printing. Also, the ambient temperature was maintained at almost 15–18 °C during the 3D printing. After fully melted, theprinting and dragging region using the mPCL was printed at theequal pneumatic pressure of 600 ± 10 kPa and used the 100- *μ*m size precision nozzles (SHN-0.1 N, Musashi, Japan). However, feed rate was adjusted that the printing region had the 4 mm/sec, while the dragging region has 150 mm/sec for creating the stretching material. The standoff distance of a vertical-direction was applied 80 *μ*m. After the mPCL MFT construct had been fabricated, mucosal and muscular bioinks were printed sequentially in the inner space of the construct. when the printed of the cell-laden bioink, the operating pneumatic pressure was used the 30 ± 10 kPa without transfer of print head and used the 26 G nozzles (PN-26G-13, U-Jin Tech, Korea). The bioprinting temperature was used 4 °C. After completion of thecell-laden bioink printing,bioink in the MFT construct was gelation in 37 °C incubator.

Prepared cells - Human esophageal epithelial cells (hEECs) and human esophageal smooth muscle cells (hESMCs) were used in this study. Both cells detached and mixed with dECM before the fabrication of the mucosa and muscle bioinks, which were then injected into the scaffold. The bioink preparation and 3Dprinting processes were conducted in ice conditions.

### Tensile testing

Tensile testing using a Universal Testing Machine (UTM; Model E42, MTS, Germany) was conducted to evaluate the ultimate strength and Young’s modulus of the fabricated MFT constructs. Five specimens were prepared for each of the four abovementioned cases (2L-NP, 2L-P, 3L-NP, and 3L-P). The length of these tensile specimens was 10 mm, with 3.25 mm at each end fixed in the UTM jig and the remaining 3.5 mm used for tensile testing. The capacity of the load cell was 5.0 kN.The fixed specimens were elongated at a speed of 0.1 mm/s. To calculate the stress, the cross-sectional area of the specimen needs to be determined. Because the cross-sectional area of the tested specimens was not rectangular or circular (for which the surface area is straightforward to calculate), it was estimated from top-view FE-SEM images of each specimen type. The cross-sectional area of the two-layered and three-layered MFT construct was calculated to be 9.21 ± 0.21 mm^2^ and 11.05 ± 0.15 mm^2^, respectively.

### Finite element analysis

To quantitatively evaluate the effect of the wrinkles on flexibility in the radial direction and the effect of the bellows shape on bending flexibility, finite element structural analysis was conducted using a commercial FEA program (ABAQUS, DassaultSystemes® SIMULIA, USA).

To simulate the radial-direction flexibility of the inner and middle layers of the MFT construct, a hollow cylindrical model with vertical-direction wrinkles was designed (Fig. 3a) to represent the inner and middle layers. The geometry of the middle layer is presented in Supplementary Fig. 6a. A simple hollow cylindrical model with an outer diameter of 4 mm, a length of 20 mm, and a thickness of 0.2 mm was also used as a numerical control model. A uniform pressure (0.2 MPa) was applied to the inner surface of the model and minimum displacement boundaries were set to prevent rigid body motion (Fig. 3a). For the finite element analysis, quadratic tetrahedron elements (the C3D8 element in ABAQUS) were used, and there were 36,002 and 113,655 nodes in the control and wrinkle models, respectively.

To simulate the bending flexibility of the outer layer of the MFT structure, a hollow cylindrical model with a bellows shape was designed (Fig. 3c) that represented the outer layer. The geometry of the outer layer is presented in Supplementary Fig. 6b. Another simple hollow cylindrical model with an outer diameter of 6 mm, a length of 20 mm, and a thickness of 0.2 mm was used as a control numerical model. A bending moment (40 N·mm) was applied to both ends of the model and minimum displacement boundary conditions were set to prevent rigid body motion (Fig. 3c). Quadratic tetrahedron elements (the C3D8 element in ABAQUS) were used in the finite element analysis, with 41,538 and 86,688 nodes in the control and bellows models, respectively. For the numerical models, Young’s modulus and Poisson’s ratio were assumed to be 400 MPa and 0.3, respectively^44,45^.

### Preparation of esophageal dECM

A porcine esophagus was collected from Pignara, a verified slaughterhouse. The esophageal mucosa and esophageal muscular tissue were isolated from the esophagus and prepared using a method that was a slightly modified version of that used in a previous study.

The esophageal mucosal tissue was cut into pieces 1–2 mm thick. The chopped esophageal mucosa was rinsed in phosphate buffered saline (PBS) and stirred into a 1% Triton X-100 solution for 72 h followed by 1% sodium dodecyl sulfate (SDS) solution for 48 h. After then, it used with a 50 U/ml nuclease solution (DNase) at 37 °C for 72 h under gentle agitation.

The esophageal muscular tissue was also cut into pieces 1–2 mm thick. The chopped esophageal muscle was rinsed in PBS and stirred into a 1% Triton X-100 solution for 12 h followed by 1% SDS in a PBS solution for 12 h. The esophageal muscular tissue was used with a 50 U/ml DNase solution at 37 °C for 24 h under gentle agitation.

The same process was employed for the decellularized mucosal and muscular esophageal tissue. The samples were rinsed using PBS for at least three days and treated with a solution of 0.1% peracetic acid in 4% ethanol for 2 hours. After that, the samples were washed again with autoclaved distilled (DI) water. The obtained dECM was lyophilized and stored at −20 °C.

### Biochemical characterization

To verify the results of the decellularization process, the residual DNA was measured. To quantify the DNA content, a Quant-iT™ PicoGreen™ dsDNA Assay Kit (Invitrogen Life Technologies, USA) was used following the manufacturer’s directions. PicoGreen solution was added to each sample, and the results were analyzed using a GenePix® 4000B microarray scanner (Molecular Devices, USA). All measurements were performed in triplicate. The remaining extracellular matrix collagen (COL) and glycosaminoglycans (GAGs) were measured using biochemical assays. The total COL content in the dECM was estimated using conventional hydroxyproline assays. The samples were measured with a spectrophotometer (Multiskan GO microplate spectrophotometer, Thermo Scientific, USA) at 540 nm and the results were quantified by referring to a standard curve generated in advance using hydroxyproline. Native tissue was also analyzed using the same protocol as a control. The total GAG content in the decellularized tissue was measured by quantifying the sulfated glycosaminoglycans using 1,9-dimethylmethylene blue solution. The samples were measured with a spectrophotometer at 525 nm. A standard curve was generated using chondroitin sulfate A in advance. Native tissue was also analyzed under the same protocol as a control.

### Rheological behavior

Before rheological testing, lyophilized dECM was digested in a solution of 0.5 M acetic acid with pepsin. The solution and esophageal dECM were mixed at a 1:10 ratio for 5 days at room temperature (pre-gel). The solution pH was initially acidic, so the pH was adjusted to be neutral using 10 M NaOH. To avoiding gelation, the experiments were conducted under ice conditions. Rheological analysis was conducted using a rheometer (Discovery HR-2; TA Instruments, USA), and all rheological measurements were performed in triplicate.

To analyze the pre-gel viscosity, a steady shear sweep from 0.1 to 1000/s was conducted on the 1.0, 1.5, and 2.0% dECM pre-gels. The viscosity measurements were taken at 4 °C. To measure the frequency-dependent storage (G’) and loss (G”) moduli of the 1.0, 1.5, and 2.0% dECM gels, dynamic frequency sweep analysis in the range of 0.1–100 rad/s was conducted at a strain of 2%. Before this experiment, the gelation process was occurred for 30 min at 37 °C. Finally, to measure the gelation kinetics, 1.0, 1.5, and 2.0%dECM pre-gels were analyzed using a temperature sweep in the range of 4–37 °C. The complex modulus (G*) was recorded as the samples were subjected to temperature ramp testing. The initial temperature was 4 °C and raised to 37 °C in increments of 5 °C/min, at which it was maintained for 40 min.

### Cell culturing

Human esophageal epithelial cells (hEECs; Het-1A, ATCC, USA) and human esophageal smooth muscle cells (hESMCs; ScienCell Research Laboratories, USA) were used in this study. The hEECs were cultured in LHC-9 medium (Gibco, USA) supplemented with 1% penicillin/streptomycin (P/S), and the hESMCs were cultured in smooth muscle cell medium (ScienCell Research Laboratories, USA) with 2% fetal bovine serum (FBS), 1% smooth muscle cell growth supplement and 1% P/S. When the hEECs were co-cultured with the hESMCs, epithelial cell medium-2 (ScienCell Research Laboratories, USA) with 1% epithelial cell growth supplement-2 and 1% P/S was used.

The hEECs and hESMCs were detached from the tissue culture plate with 0.25% trypsin-EDTA and centrifuged at 1,000 rpm for 3 min. The concentration of cells used in this study was 3.5 ×10^6^ cells/ml. The hEECs and hESMCs were mixed with esophageal mucosal-derived decellularized extracellular matrix (emuc-dECM) and esophageal muscular-derived decellularized extracellular matrix (ems-dECM) pre-gel at the desired concentration, respectively. The bioinks were cross-linked at 37 °C for at least 30 min in an incubator condition.

### Study of cell behavior in the MFT structure with bioinks

After the fabrication of the multilayered free-form 3D cell-printed tubular construct with decellularized inner and outer esophageal tissue-derived bioinks, a number of *in vitro* and *in vivo* studies were performed.

For the cell proliferation assays, Cell Counting Kit-8 (CCK-8; Dojindo, Japan) assays were conducted following the manufacturer’s protocol. CCK-8 solution was added to each sample at a 1:10 ratio and incubated for 4 h. The results were analyzed using a spectrophotometer (Multiskan GO microplate spectrophotometer; Thermo Scientific, USA) at an optical density of 450 nm. All measurements were taken in triplicate.

To test cell viability, the MFT construct with bioinks was stained using a LIVE/DEAD Viability/Cytotoxicity Kit (Invitrogen Life Technologies, USA) following the manufacturer’s protocol. Ethidium homodimers and calcein-AM were mixed at a ratio of 2:1 in PBS and added to each sample for 1 hour in an incubator condition. The results were observed using fluorescence microscopy (DM 750; Leica Microsystems, Germany).

The cells were stained with Phalloidin-Tetramethylrhodamine B isothiocyanate (Phalloidin; Sigma Aldrich, USA) to visualize the actin cytoskeleton, while they were stained with Hoechst 33342 (Hoechst; Thermo Fisher Scientific, USA) to visualize the cell nuclei. The results were prepared for histological analysis by placing them in 4% paraformaldehyde (PFA) for overnight. The cells were permeabilized with 0.1% Triton X-100 and washed in PBS. Cells were then stained with a 50 mg/ml phalloidin solution in PBS for 1 h at room temperature and washed with PBS before the addition of 0.1 μg/ml Hoechst working solution at a 1:10,000 dilution for 20 min. Phalloidin- and Hoechst-stained images were captured and merged using confocal microscopy (Leica Microsystems, USA). The Image J program (National Institutes of Health, USA) was used to analyze and present the cell morphology.

### Analysis of the gene expression

We fabricated the MFT construct+esophageal bioinks and MFT construct+collagen bioinks cultured for 7 days in an incubator. MFT construct+esophageal bioinks means hEECs-laden emuc-dECM bioink (eMFT+dECM) in the inner layer and hESMCs-laden ems-dECM bioink (sMFT+dECM) in the outer layer. MFT construct+collagen bioinks means hEEC-laden collagen bioink (eMFT+col) in the inner layer and hESMCs-laden collagen bioink (sMFT+col) in the outer layer. All bioinks have the same concentration at 1.5%.

Total RNAs were isolated from each sample by using RNA isoplus (TaKaRa, Japan). 1 μg of the RNA was synthesized into the cDNA using a Maxima First Strand cDNA synthesis kit (Thermo Scientific, USA). Quantitative real time polymerase chain reaction (qRT-PCR) was performed with SYBR® Green PCR Master Mix (Applied Biosystems, USA), and an ABI 7500 Real-time PCR system (Applied Biosystems, USA). PCRconditions were 40 cycles of 95 °C for 15 s, 60 °C for 1 m. The following primers were used that alpha-smooth muscle actin (α-SMA)^46^, E-cadherin^47^, and glyceraldehyde 3-phosphate dehydrogenase (GAPDH)^48^. The sequences of primer sets used in this analysis were shown in Supplementary Table 1. Fold change in the expression level of each gene were calculated for each treatment group using CT values normalized to transcript levels of the GAPDH^49^.

### Animals

Standard laboratory rats (Rattusnorvegicus) were individually housed in wire bottom cages in a temperature- and light-controlled room. All animals were allowed to acclimate to the housing facility for 5–7 days prior to intervention and had ad libitum access to food and water. Animal care, housing, and procedures were performed in accordance with the protocol approved by the Animal Care and Use Committee of Pohang University of Science and Technology, South Korea (POSTECH-2015-0069).

### Statistical analysis

The data are expressed as the mean±standard deviation. All statistical analyses were conducted using GraphPad Prism (GraphPad software; La Jolla, CA). All experiments were performed in triplicate. Statistical significance was determined using two-tailed t-tests. Statistical significance was set at *p < 0.05, **p < 0.005, ***p < 0.0005, and ****p < 0.0001.

## Supplementary information

Supplementray data.

Supplementary video 1.

Supplementary video 2.
