# Supplementary material for: Multi-layered Free-form 3D Cell-printed Tubular Construct with Decellularized Inner and Outer Esophageal Tissue-derived Bioinks
Source: Sci Rep. 2020 Apr 29;10:7255. doi: 10.1038/s41598-020-64049-6 (PMC7190629; doi:10.1038/s41598-020-64049-6)
Supplement: Supplementary file 1 — Supplementray data. [file 41598_2020_64049_MOESM1_ESM.docx]

**Supplementary Data**

**Multi-layered Free-form 3D Cell-printed Tubular Construct with Decellularized Inner and Outer Esophageal Tissue-derived Bioinks**

Hyoryung Nam^1,#^, Hun-Jin Jeong^2,#^, Yeonggwon Jo^3^, Jae Yeon Lee^4^, Dong-Heon Ha^4^, Ji Hyun Kim^5^, Jae Hee Chung^5^, Young-Sam Cho^2,6^, Dong-Woo Cho^4^, Seung-Jae Lee^2,6,*^, Jinah Jang^1,3,4,*^

^1^ Department of Creative IT Engineering, Pohang University of Science and Technology, San 31, Pohang, Gyeongbuk, Republic of Korea.

^2^ Department of Mechanical Engineering, Wonkwang University, Iksan-daero, Iksan, Jeollabuk-do, Republic of Korea.

^3^ School of Interdisciplinary Bioscience and Bioengineering, Pohang University of Science and Technology, San 31, Pohang, Gyeongbuk, Republic of Korea.

^4^ Department of Mechanical Engineering, Pohang University of Science and Technology, San 31, Pohang, Gyeongbuk, Republic of Korea.

^5^ Department of Surgery, Collage of Medicine, The Catholic University of Korea, Banpo-daero, Seoul, Republic of Korea.

^6^ Department of Mechanical and Design Engineering, Wonkwang University, Iksan-daero, Iksan, Jeollabuk-do, Republic of Korea.

^#^ These authors contributed equally to this work.

^*^ co-correspondence to S.-J. L. ([sjlee411@wku.ac.kr](mailto:sjlee411@wku.ac.kr)) and J.J. ([jinahjang@postech.ac.kr](mailto:jinahjang@postech.ac.kr))

**
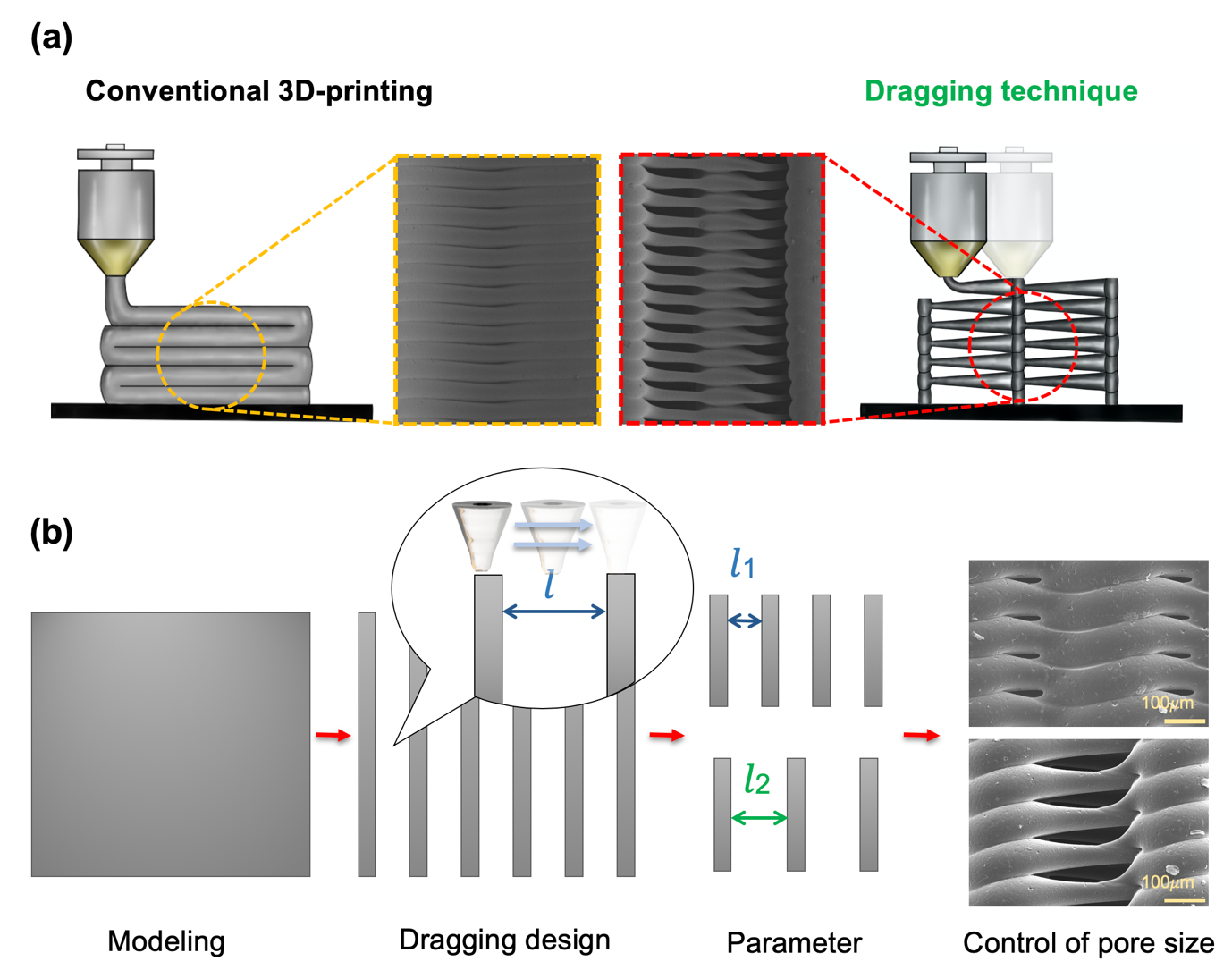
**

**Supplementary Fig. 1** (a) Comparison of the conventional 3D printing and dragging technique and (b) schematics of the dragging technique mechanism

**
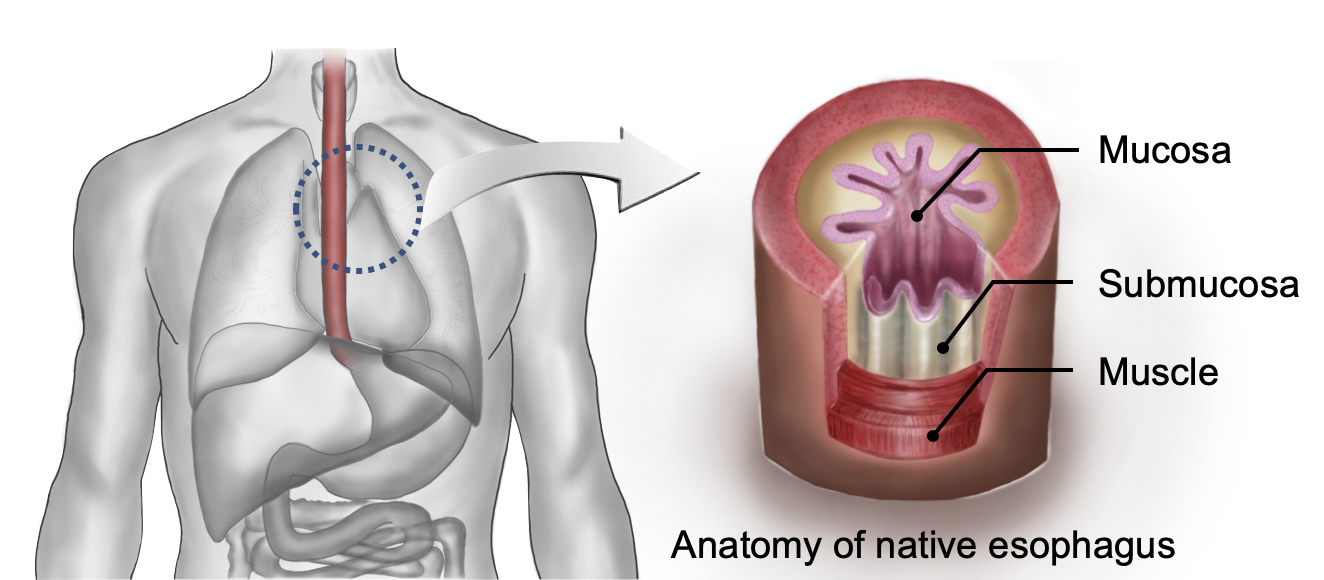
**

**Supplementary Fig. 2** Schematic of the native esophagus.

**
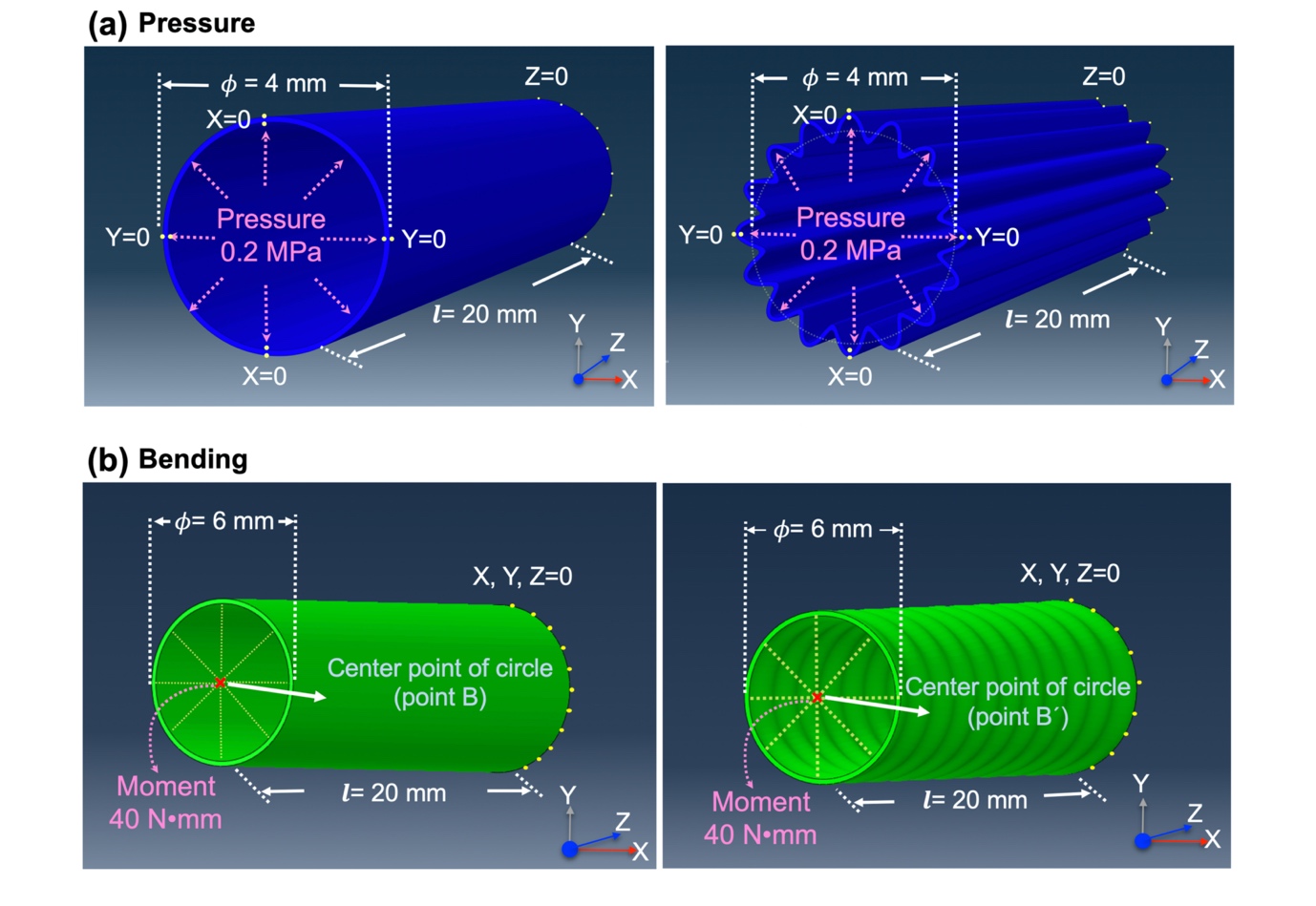
**

**Supplementary Fig. 3** Mechanical property analysis of the esophageal construct (a) Boundary condition of hollow cylindrical model with wrinkle shape (like inner and middle layers in MFT construct), (b) bellows shape (like outer layer) and control model for FEA analysis.


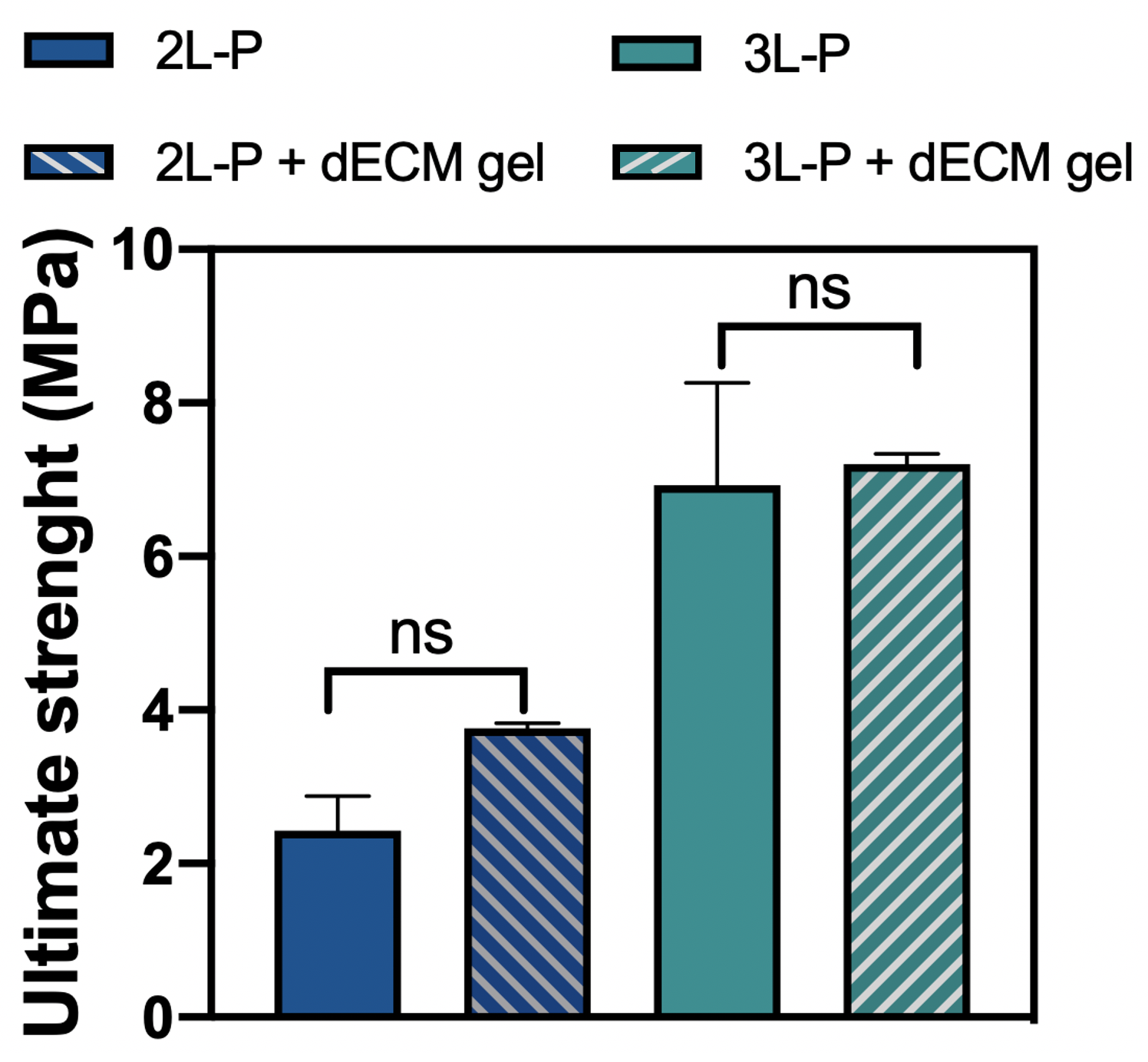


**Supplementary Fig. 4** Comparison of ultimate strength the of the MFT construct with or without dECM gel (without cells).

**
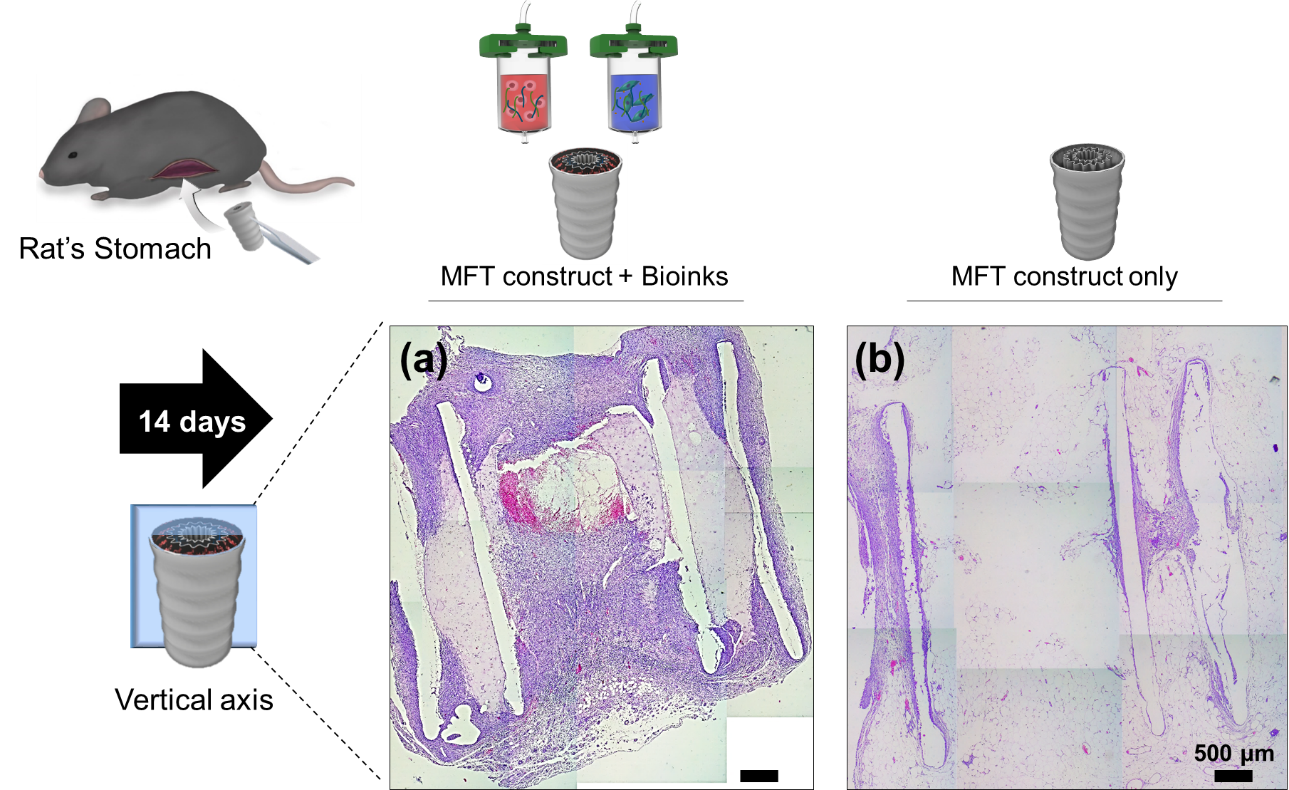
**

**Supplementary Fig. 5** *In vivo* maturation of the MFT constructs and H&E histology results after 14 days. (a) MFT construct + esophageal tissue-derived bioinks (b) MFT construct only.

**Material & method**

***In vivo* maturation of the construct (a) MFT construct + esophageal tissue-derived bioinks (b) MFT construct only.**

A total of 2 male standard laboratory rats (Rattus norvegicus, 8 weeks-old) were distributed into two experimental groups: Group A, MFT construct with emuc-dECM + hEECs in the inner layer, ems-dECM + hESMCs in the outer layer (MFT construct + Bioinks); Group B, MFT construct without bioinks (MFT construct only).

The animals were then taken to the operating room under general anesthesia and sterile conditions. The animals were anesthetized with a Zoletil (15 mL/kg)–Rompun (0.5 mL/kg) mixture and each rat was implanted with two respective samples at abdominal subcutaneous. All rats were recovered from anesthesia and treated daily with Tramdol (3 mg/kg) for analgesia and bacitracin ointment for wound care. All animals were monitored daily for 14 days, and there were no adverse events noted in any of the animals. All rats were sacrificed at 2 weeks, and the samples retrieved from these rats were dedicated for histological analysis.

The harvested constructs were prepared histologically that put in 4 % PFA for at least 24 h, then further processed for paraffin embedment and H&E staining. The results observed by a bright-field microscopes (Eclipse Ti; Nikon, Japan).

**
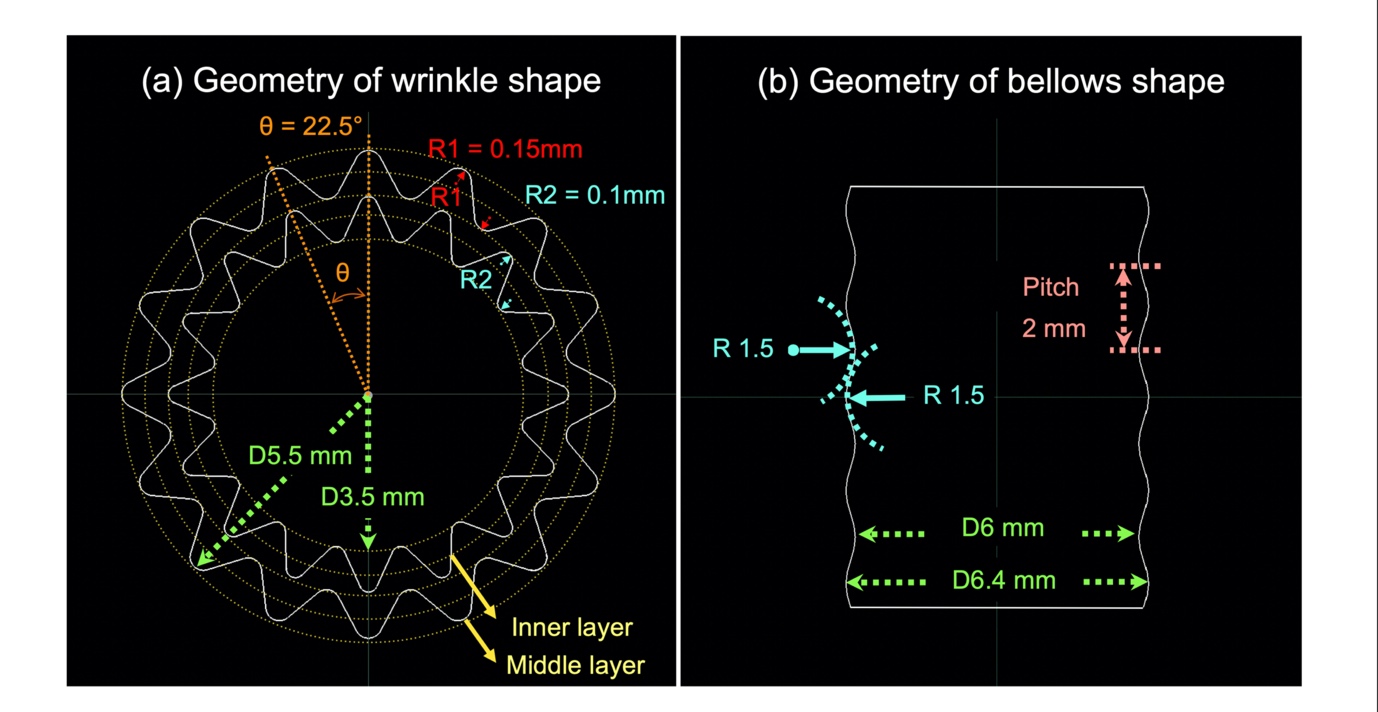
**

**Supplementary Fig. 6** Geometric of inner, middle layer (wrinkle) and outer layer (Bellows).

**Material & method**

**Geometrical information of the multilayered free-form tubular (MFT) construct**

The inner and middle layers in the MTS construct was designed with vertical-direction wrinkle shape. (**Fig. S1 (a)**) The wrinkle shape of the middle layer was designed to have an outer radius, a center radius, and an inner radius of 5.5 mm, 5 mm, 4.5 mm, respectively. The radius of curvature of the wrinkle (middle layer) peaks and valleys is 0.15 mm (R1), and the intervals between the peaks to peaks (pitch) are 22.5 degrees. The wrinkle shape of inner layer was designed to have an outer radius, a center radius, and an inner radius of 4.5 mm, 4 mm, 3.5 mm, respectively. The pitch degree is the same as the middle layer, and the radius of curvature of the peak and valley is 0.1 mm.

The outer layers in the MTS construct was designed with bellows shape. (**Fig. S1 (b)**). In the cross-section view of vertical direction, the bellows shape has a wavy pattern with a radius of curvature of 1.5 mm, and the pitch distance is 2 mm. Also, the hollow cylindrical MFT construct of outer layer has an inner diameter of 6 mm and an outer diameter of 6.4 mm.

**Supplementary Table 1.** Primer sequences for α-SMA, E-cadherin, and GAPDH.

| Gene | Sequence (5′–3′) | |
| --- | --- | --- |
| α-SMA | Forward | TGAAGAGCATCCCACCCT |
|  | Reverse | ACGAAGGAATAGCCACGC |
| E-cadherin | Forward | GAACAGCACGTACACAGCCCT |
|  | Reverse | GCAGAAGTGTCCCTGTTCCAG |
| GAPDH | Forward | CAATGACCCCTTCATTGACC |
|  | Reverse | GACAAGCTTCCCGTTCTCAG |

**SUPPLEMENTARY Video 1** 3D bioprinting process of the biomimetic esophageal construct using the dragging technique.

**SUPPLEMENTARY Video 2** Flexibility and recovery test of 3-layer pore construct using radial-direction compression and bending motion.
